# Olean-12-Eno[2,3-c] [1,2,5]Oxadiazol-28-Oic Acid (OEOA) Induces G_1_ Cell Cycle Arrest and Differentiation in Human Leukemia Cell Lines

**DOI:** 10.1371/journal.pone.0063580

**Published:** 2013-05-16

**Authors:** Yu Pong Ng, Yuewen Chen, Yueqing Hu, Fanny C. F. Ip, Nancy Y. Ip

**Affiliations:** 1 Division of Life Science, The Hong Kong University of Science and Technology, Clear Water Bay, Hong Kong, China; 2 State Key Laboratory of Molecular Neuroscience, The Hong Kong University of Science and Technology, Clear Water Bay, Hong Kong, China; 3 JNU-HKUST Joint Lab, Ji-Nan University, Guangzhou, Guang Dong, China; Southern Illinois University School of Medicine, United States of America

## Abstract

Oleanolic acid (3β-hydroxy-olea-12-en-28-oic acid) is a natural pentacyclic triterpenoic acid found in many fruits, herbs and medicinal plants. In the past decade, increasing evidence has suggested that oleanolic acid exhibits inhibitory activities against different types of cancer including skin cancer and colon cancer, but not leukemia. We report here that a derivative of oleanolic acid, olean-12-eno[2,3-c] [1], [2], [5]oxadiazol-28-oic acid (designated OEOA) effectively blocks the proliferation of human leukemia cells. OEOA significantly reduces cell proliferation without inducing cell death in three types of leukemia cell lines, including K562, HEL and Jurket. Moreover, exposure of K562 cells to OEOA results in G_1_ cell cycle arrest, with a concomitant induction of cyclin-dependent kinase inhibitor p27 and downregulation of cyclins and Cdks that are essential for cell cycle progression. Interestingly, OEOA also enhances erythroid differentiation in K562 cells through suppressing the expression of Bcr-Abl and phosphorylation of Erk1/2. These findings identify a novel chemical entity for further development as therapeutics against leukemia.

## Introduction

Leukemia is a malignant disease which broadly covers a number of cancers of the blood, bone marrow, and lymphoid systems. Based on how the disease develops (acute or chronic) and the blood cells affected (lymphocytes or myelocytes), they are categorized into four main types, i.e. acute lymphocytic leukemia (ALL), chronic lymphocytic leukemia (CLL), acute myelocytic leukemia (AML), and chronic myelocytic leukemia (CML) [1]–[4].

While leukemia affects both adults and children of both genders with most cases (>90%) diagnosed in adults, leukemia is the most common form of cancer in children and adolescents, accounting for about one third of cancers in individuals aged under 20. The United States National Cancer Institute estimates that the number of new cases is 47,150 in 2012 and about 23,540 people will die from leukemia this year [5]. The current therapeutic treatments include anti-cancer medication such as imatinib (Gleevec®), chemotherapy, radiation therapy, stem cell transplant, and in some cases, surgical removal of the spleen [1]–[4]. The choice of treatment depends on the type of leukemia, as well as the health and age of the patient.

Despite substantial progress in current and emerging treatment strategies, short remission duration has been reported [1]–[4]. The development of drug resistance, especially to the first treatment, or in the first or subsequent relapses, presents a big challenge for drug development [6]–[9]. Furthermore, most of the chemotherapeutic anti-cancer agents work on a non-targeted basis and induce various degrees of side effects such as fatigue, muscle and joint pain, impaired immune responses, anemia, neutropenia and thrombocytopenia [1]–[4], [6]–[9]. Therefore, the search for new anticancer agents for leukemia patients is of paramount importance.

Oleanolic acid (3β-hydroxy-olea-12-en-28-oic acid, OA) is a natural pentacyclic triterpenoic acid [10], [11]. In the past decade, an increasing number of studies have reported a wide range of pharmacological activities of OA including anti-inflammatory, anti-cancer, anti-HIV, and hepato-protective effects [12], [13]. While OA effectively suppresses a number of tumors [12]–[19], the compound is ineffective in inhibiting leukemia cell proliferation [14], [19], [20]. In the present study, we report the characterization of an OA derivative, olean-12-eno[2,3-c] [1], [2], [5]oxadiazol-28-oic acid (OEOA), which displays anti-leukemia properties.

## Materials and Methods

### Preparation of OEOA

**Figure 1:**
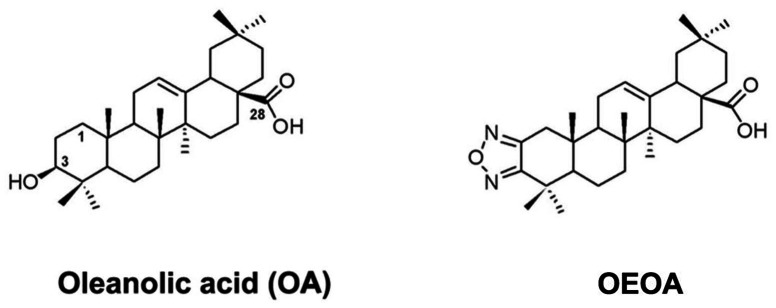
Structures of oleanolic acid and OEOA. Oleanolic acid, 3β-hydroxy-olea-12-en-28-oic acid, MW: 456.71; OEOA, olean-12-eno[2,3-c] [1], [2], [5]oxadiazol-28-oic acid, MW: 480.69.

Oleanolic acid (OA) was purchased from Sigma-Aldrich (St. Louis, MO, USA). OEOA was synthesized from OA as previously described [21]. The chemical structure of the compound was determined by ^1^H-NMR, ^13^C-NMR and mass spectrometry as illustrated in Fig. 1. The purity was confirmed to be over 99% by HPLC-ELSD analysis. Stock solution was prepared in dimethyl sulfoxide (DMSO, Sigma-Aldrich) and stored at -80°C.

### Cell Cultures

Human erytholeukemia cell lines, K562 and HEL and a T-cell leukemia cell line, Jurket (American Type Culture Collection, ATCC, MD, USA) were maintained in RPMI 1640 medium (Invitrogen, Carlsbad, CA, USA) supplemented with 10% fetal bovine serum (FBS, Invitrogen). HepG2 (hepatocellular liver carcinoma), MCF-7 (breast adenocarcinoma), and HeLa (epithelial cervical cancer) cells were cultured in DMEM (Invitrogen) supplemented with 10% FBS. The cell cultures were incubated at 37°C with 5% CO_2_ in humidified air. Human neonatal keratinocytes (HEKneo) were purchased from Invitrogen. HEKneo cells were cultured using Epilife with 1% HKGS in a T75 flask (BD Biosciences, San Jose, CA, USA). The cultures were maintained at 37°C in a humidified incubator with 5% CO_2_, following the manufacturer’s protocol.

### Cell Proliferation and Viability Assays

Cells were seeded on a 96-well plate at the density of 3×10^4^ per well and then incubated overnight at 37°C with 5% CO_2_. Following treatment of cells with OEOA or DMSO (both diluted in culture medium) for 48 h, MTT (3-[4,5-dimethylthiazol-2-yl]-2,5-diphenyltetrazolium bromide) assay was performed to measure the cell viability (USB, Cleveland, OH, USA). To examine the cytotoxic effect of OEOA, K562 and HEL cells were seeded onto a 6-well plate at an initial cell density of 1×10^5^ per well. Following incubation at 37°C with 5% CO_2_ overnight, cells were then treated with OEOA (1 µM) or DMSO for 6 days. Viable cells were counted daily after being stained with 0.4% trypan blue (Sigma Aldrich).

### Cell Cycle Analysis

K562 cells (1×10^5^ per well in a 6-well plate) were treated with OEOA for 24 h. Cells were harvested and washed twice with ice cold PBS with 1% calf serum (Invitrogen). The cell pellets were then resuspended in 100 µl of PBS with 1% calf serum, fixed with 80% ethanol at 4°C for 1 h, resuspended and incubated in 500 µl of Tris-EDTA solution containing 40 µg/ml propidium iodide (PI) and 40 µg/ml of RNaseA at 37°C for 30 min [22]. Cells were then analyzed by flow cytometry (Becton-Dickinson, CA, USA). Approximately 10,000 cells were counted for each sample. The percentage of cell distribution was calculated using Cell Quest software.

### Western Blot Analysis

Following treatment with OEOA, cells were harvested and lysed with RIPA buffer containing 150 mM NaCl, 1% Nonidet P-40, 1 mM EDTA, 0.5% deoxycholic acid, 2 µg/ml aprotinin, 1 mM PMSF, 5 mM benzamidine, 1 mM sodium orthovanadate and 10 µg/ml soybean trypsin inhibitor in 50 mM Tris buffer, pH 7.4. Protein concentration was determined by Bio-Rad protein assay (Hercules, CA, USA). Equal amounts of proteins were resolved by SDS electrophoresis, and then transferred to nitrocellulose membranes for immunoblotting. Primary antibodies against phospho Ser807/811-retinoblastoma protein (p-Rb), phospho-Erk1/2 (p-Erk1/2), Rb, Erk1/2, secondary antibodies (HRP-conjugated goat anti-mouse, anti-rabbit antibodies) were purchased from Cell Signaling Technology (Beverly, MA, USA), and an antibody against actin was from Sigma-Aldrich. Antibodies specific for p27, Cyclin D1, Cyclin E, Cdk4, Cdk6 and c-Abl were obtained from Santa Cruz Biotechnology (Santa Cruz, CA, USA). The polyclonal primary antibody against retinoic acid-regulated nuclear matrix-associated protein (RAMP) was generated as described previously [23]. Immunoprecipitation of Bcr-Abl from cell lysates was performed using c-Abl antibody. The immunoprecipitates were collected with protein G sepharose beads (GE Healthcare, Pittsburgh, PA, USA), and analyzed by Western blotting using 4G10 anti-phosphotyrosine antibody (Millipore Billerica, MA, USA). Proteins on the blots were detected by an enhanced chemiluminescence (ECL) detection system (Invitrogen). Quantification of the Western blots was performed using ImageJ (http://rsbweb.nih.gov/ij/).

### Reverse Transcription and Real-time Polymerase Chain Reaction

Total RNA was extracted using Purelink micro-to-midi total RNA purification system (Invitrogen). Five microgram total RNA of each sample was reverse-transcribed using oligo (dT) primers and SuperScript II reverse transcriptase (Invitrogen) in 20-µl volume. Quantification of the target genes was performed with Power SYBR Green PCR master mix kit in 7500 Fast-real time PCR system, according to the manufacturer’s instructions (Applied Biosystems, Foster City, CA). The specificity of the SYBR Green PCR signal was confirmed by melting curve analysis. Following primer sequences were used: *γ-globin* forward primer: 5′-TGGCAAGAAGGTGCTGACTTC-3′; γ*-globin* reverse primer: 5′-TCACTCAGCTGGGCAAAGG-3′ [24]; *cd41b* forward primer 5′-GCTGCAGATGGACGCAGCCA-3′; *cd41b* reverse primer 5′-GCATGTAGTGGGCGCCCTGG-3′. In each experiment, human hypoxanthine phosphoribosyltransferase 1 (*hprt1*? mRNA was used as an endogenous reference with the primer sequences as follows: forward primer: 5′-TGACACTGGTAAAACAATGCA-3′; reverse primer: 5′-GGTCCTTTTCACCAGCAAGCT-3′ .

### Pharmacokinetic Studies for OA and OEOA

To study the cellular permeability of OEOA and OA in K562 cells, culture medium and cell lysates were collected at different time intervals after incubation. K562 cells (3×10^5^ in a T25 flask) were treated with 1 µM of OA or OEOA, and cell pellets were collected by centrifugation. The culture medium (3 ml) was aspirated and extracted with equal volume of ethyl acetate for two times (30 min each). After combining the ethyl acetate extracts, the samples were vacuum-dried and dissolved in methanol for detection. Cell pellets were washed with cold phosphate-buffered saline and lysed in RIPA buffer. The cell lysates were dried in vacuum and dissolved in methanol for detection. Area under peak (AUC) was obtained and percentage of compounds in cell lysates was calculated as: % of the compound (cell lysate) = AUC (cell lysate)/AUC (medium+cell lysate) x 100%. The remaining samples were then loaded onto the preparative HPLC and the corresponding fractions of OA or OEOA were collected and dried. They were dissolved with methanol-d6 in NMR tube, and then analyzed in the Varian 300 NMR to confirm the identity of compound.

To examine the concentrations of OEOA and OA *in vivo*, male C57Bl/6 mice (12-week-old) were obtained from Animal Care Facility in the Hong Kong University of Science and Technology (HKUST). OA and OEOA were dissolved in a solution of 3% dimethylacetamide/10% Tween-80 in water (3∶10∶87 by vol.) and administered intraperitoneally to the mice at 20 µmol/kg (injection volume 10 ml/kg). The procedures in this study were approved by HKUST Animal Ethics Committee and conducted in accordance with the Code of Practice and Use of Animals for Experimental Purpose. Terminal blood (K_2_EDTA tubes, 300 µl) was collected at indicated time points after OA and OEOA administration. Plasma was obtained by centrifuging the blood at 3,800×g for 10 min at 4°C followed by extraction with ethyl acetate twice and pooled extracts were evaporated to dryness. The sample was then dissolved with 0.1 ml methanol, vortexed for 30 s followed by ultrasonication for 5 min and centrifuged at 17,900×g for 5 min. The supernatant was transferred into vial for high performance liquid chromatography tandem mass spectrometry analysis (HPLC-MS/MS).

OA and OEOA were analyzed by an Agilent 1200 series HPLC system coupled to an AB SCIEX 4000+ triple quadrupole system equipped with a turbo V source with an electrospray ionization (ESI) probe. The compounds in methanol were separated on a C18 column (Agilent Zorbax, 50×3.0 mm, 1.8 µm, 40°C) with the mobile phase consisted of (A) 0.1% formic acid in water and (B) acetonitrile. Five microliters of sample was injected and eluted by the following program at the flow rate of 0.5 ml/min: 0–7 min, 70∼100% B; 7–10 min, 100% B; 10–10.1 min, 100∼70% B; 10.1–15 min, 80% B. Detection of OA and OEOA was carried out in negative ionization mode. The ESI conditions were as follows: declustering potential −50V, entrance potential −10V, collision cell exit potential −15V, collision energies −40 V, curtain gas 20 (arbitrary units), collision gas 5 (arbitrary units), ion spray voltage −4500 kV, source temperature 350°C, ion source gas 1∶40 (arbitrary units), ion source gas 2∶10 (arbitrary units). Multiple-reaction-monitoring was used to measure OEOA and OA in samples. Standard solutions of OA and OEOA (0.2 to 20 µM) were prepared in methanol and added to mouse plasma.

### Statistical Analysis

Data are expressed as mean ± SEM of three independent experiments and analyzed by Student’s *t*-test. Results were considered statistically significant with *p*-value <0.05.

## Results

### OEOA Inhibited the Proliferation of Leukemia Cells

**Figure 2:**
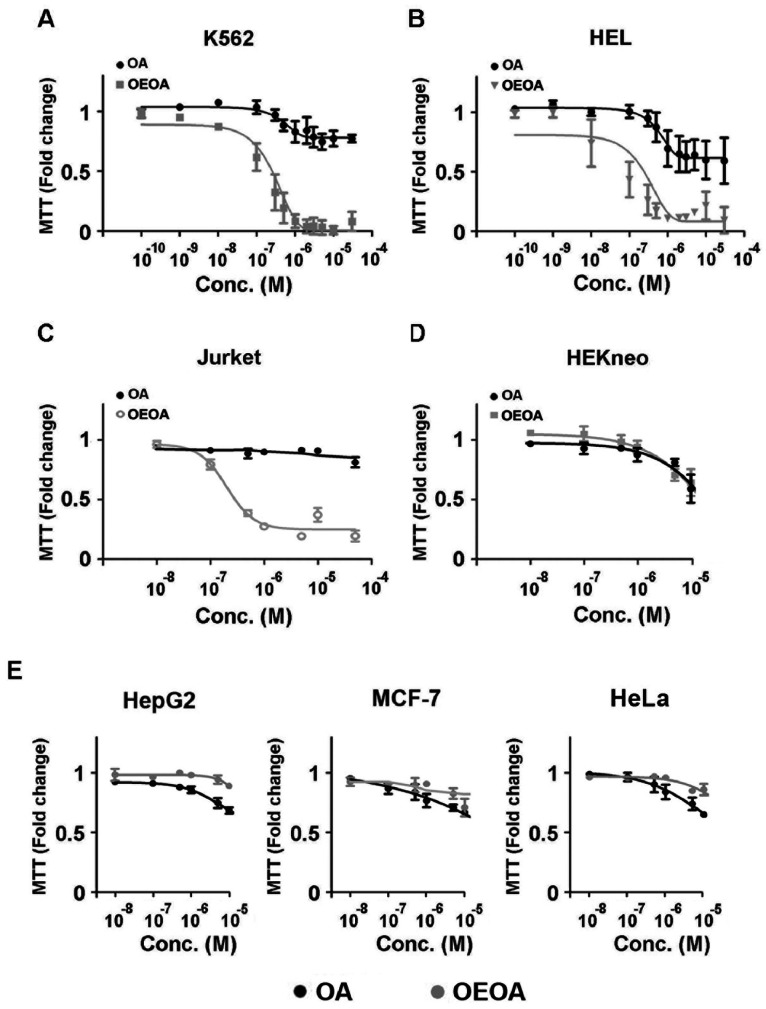
OEOA inhibited cell proliferation in leukemia cell lines. Different cell lines were treated with various concentrations of OEOA or OA for 2 days. Cell growth was measured by MTT assay: (A) K562, (B) HEL, (C) Jurket (D) HEKneo, and (E) HepG2, MCF**-**7 and HeLa cells. Data are mean ± SEM of three independent experiments (* *p*<0.05).

Treatment with OEOA inhibited cell proliferation of K562 cells (IC_50_ = 0.78±0.037 µM) and HEL cells (IC_50_ = 0.21±0.041 µM) in a dose-dependent manner, whereas OA showed minimal inhibitory effect on the cell growth of these two cell lines (Fig. 2A & B). OEOA similarly inhibited the growth of Jurket cells (Fig. 2C, IC_50_ = 0.29±0.025 µM). Importantly, OEOA did not suppress the growth of primary human neonatal keratinocytes (HEKneo) at a concentration up to 10 µM (Fig. 2D). OEOA also did not show any significant growth inhibition in MCF-7 (a human breast adenocarcinoma) and HeLa (a human epithelial cervical cancer) cells (Fig. 2E). Only a slight inhibition on the cell growth of a human hepatocellular liver carcinoma, HepG2, was observed at 10 µM (Fig. 2E). These results suggest the selectivity of OEOA to leukemia cells.

**Figure 3:**
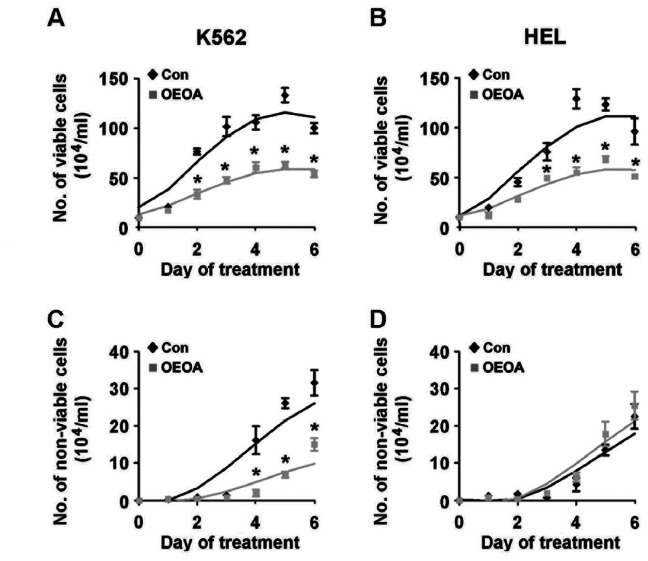
OEOA did not induce cell death in K562 and HEL cells. K562 (A & C) and HEL (B & D) cells were treated with OEOA (1 µM) for 6 days. Cell viability was measured by trypan blue exclusion as described in Materials and Methods. Data are mean ± SEM of three independent experiments (* *p*<0.05).

To examine whether the growth inhibitory effect of OEOA on the leukemia cell lines is attributable to cell death, we counted the number of viable cells after trypan blue staining. The cell growth of K562 and HEL cells was monitored for 6 days in the presence of OEOA. Treatment with OEOA resulted in a significant reduction in the number of viable cells in K562 and HEL cells (Fig. 3A & B) without significantly inducing cell death when compared to the vehicle control (Fig. 3C & D).

### OEOA Induced G1 Cell Cycle Arrest in K562 Cells

**Figure 4:**
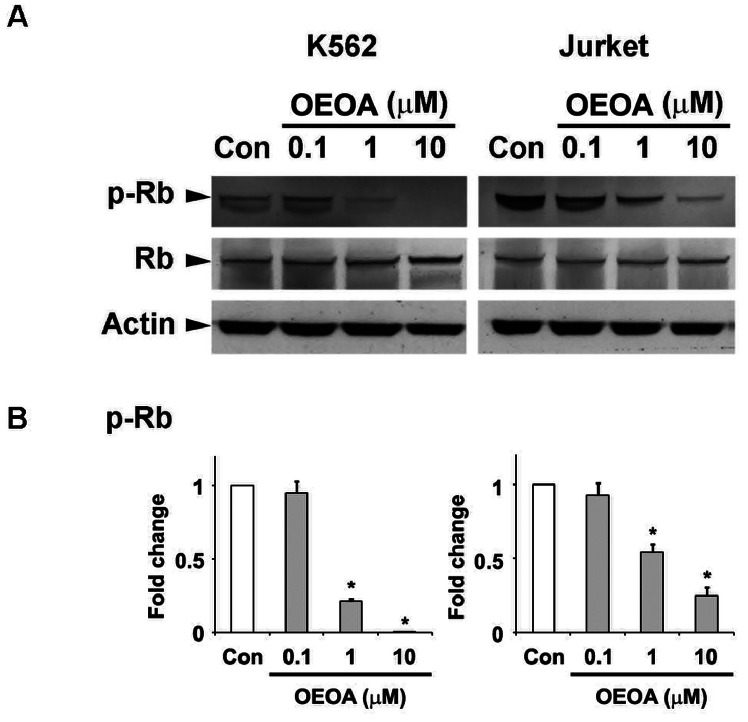
OEOA attenuated phosphorylation of Rb protein in K562 and Jurket cells. (A) K562 and Jurket cells were treated with OEOA (0.1–10 µM) for 2 days, and the cell lysates were subjected to Western blot analysis for p-Rb and Rb. Actin served as an equal loading control. Histograms in (B) show the relative expression of p-Rb (normalized to actin) as compared to the vehicle-treated cells. Results were representative blots from three separate experiments, (* *p*<0.05).

**Figure 5:**
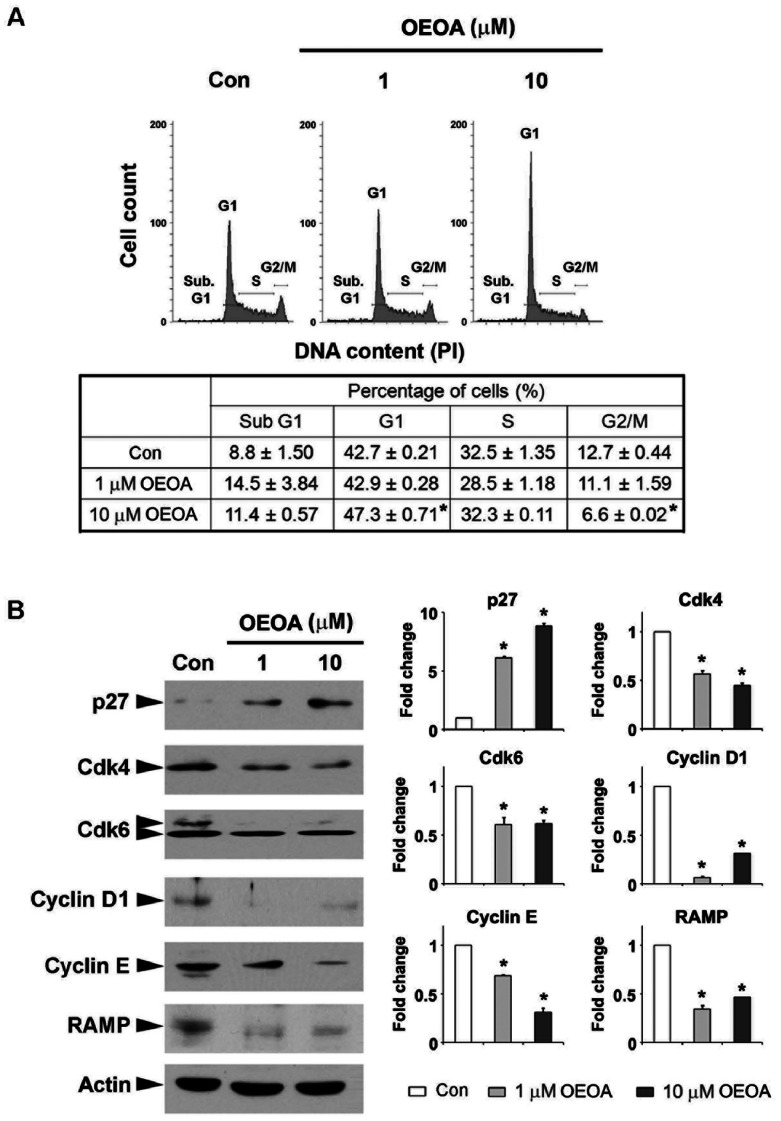
OEOA induced G1 cell cycle arrest in K562 cells. (A) Cells were incubated with OEOA (1 or 10 µM) for 24 h. The distribution of cell cycle was examined by PI staining method. The table summarized the distribution of cells in OEOA-treated or control cells. Data represented mean ± SEM of three independent experiments (* *p*<0.05). (B) K562 cells were cultured in the presence of OEOA (1 or 10 µM) for 2 days. Total proteins were collected for Western blot analysis to detect the expression of p27, Cdk4, Cdk6, Cyclin D1, Cyclin E and RAMP. Actin served as an equal loading control. Histograms on the right show the relative expression of various proteins (normalized to actin) as compared to the control cells. Results were representative blots from three separate experiments, (* *p*<0.05).

Since OEOA inhibited the leukemia cell proliferation without eliciting cytotoxic effect, we ask whether OEOA regulates the cell cycle progression. The phosphorylation/dephosphorylation of Rb protein has been reported to regulate cell proliferation by controlling the transition from G1 to S phase [25]–[27], and hyperphosphorylation of Rb leads to the uncontrolled cell proliferation in various human cancers including leukemia [27]–[29]. We first examined the level of Rb phosphorylation in K562 and Jurket cells after OEOA treatment. We found that OEOA significantly reduced the phosphorylation of Rb protein in these two leukemia cells (Fig. 4A & B). Interestingly, analysis of OEOA-treated K562 cells by flow cytometry revealed that the percentage of sub G1 and G1 phase cells increased upon treatment with OEOA, concomitant with a reduction of the cell population in S phase and G2/M phase (Fig. 5A).

To further understand the molecular events underlying the observed G1 arrest, we next examined the effects of OEOA on key regulatory molecules including Cyclins D1/E and Cdk4/6 which co-operate to promote the transition from G1 to S phase. Expression of Cdk4 and Cdk6 and the regulatory subunit Cyclin D1 decreased in OEOA-treated K562 cells (Fig. 5B). The level of Cyclin E, which is important for activating Cdk2 during the G1 to S transition, was also reduced in response to OEOA, whereas the expression of a Cdk inhibitor, p27, was increased (Fig. 5B). Furthermore, the expression of RAMP, a component of the DDB1-CUL4-X-box E3 ubiquitin ligase complex that also required for cell cycle control [30]–[33] was attenuated following treatment of cells with OEOA (Fig. 5B), consistent with the notion that OEOA could induce G1 cell cycle arrest and concomitant modulation of the expression of cell cycle regulators.

### OEOA Promoted Erythroid Differentiation in K562 Cells

**Figure 6:**
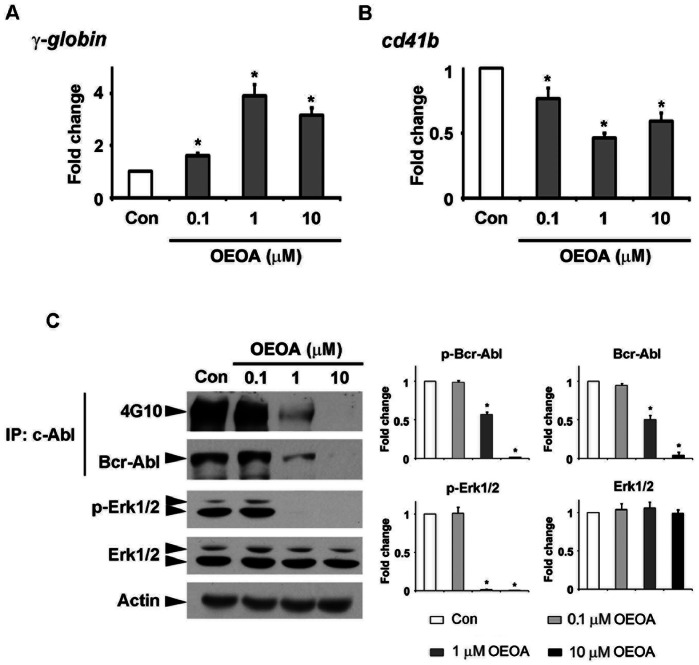
OEOA promoted erythroid differentiation in K562 cells. K562 cells were treated with OEOA (0.1–10 µM) for 2 days. Total RNA was reverse transcribed and subjected to real time-PCR analysis with primers specific to *γ-globin* (A) and *cd41b* (B), respectively. *hprt1* served as an internal housekeeping gene control. Data were expressed as fold change to the control cells as mean ± SEM of three independent experiments (* *p*<0.05). (C) K562 cells were treated with OEOA (0.1–10 µM) for 2 days. Western blot analysis of Bcr-Abl and Erk1/2 was performed. Actin served as an equal loading control. Histograms on the right show the relative expression of various proteins (normalized to actin) as compared to the control cells. Results were representative blots from three separate experiments, (* *p*<0.05).

K562 cells can be differentiated into erythroid cells, which are marked by the increased expression of embryonic/fetal globin genes, such as *ζ-, ε-*, and *γ-globin* genes [24], [34]. It has also been suggested that K562 cells can be differentiated into megakaryocytic lineage with the expression of a surface marker, CD41b [35]. We therefore examined whether OEOA enhanced the differentiation of K562 cells. Using real-time PCR analysis, we found that OEOA treatment increased the expression of *γ-globin* gene, and reduced the expression of *cd41b* (Fig. 6A & B).

Inhibition of the fusion oncogene Bcr-Abl induces erythroid differentiation of CML cell lines [35], [36]. In order to elucidate the possible mechanism by which OEOA promoted erythroid differentiation, we examined the phosphorylation and protein expression of Bcr-Abl which has been reported to negatively regulate the erythroid differentiation of CML cell lines [35], [36]. Treatment with OEOA led to the down-regulation of both total and phosphorylated Bcr-Abl levels (Fig. 6C). In addition, treatment with OEOA markedly attenuated phosphorylation of Erk1/2 (Fig. 6C) and induced the expression of p27 (Fig. 5B), both of which have been implicated in promoting erythroid differentiation [36]–[39]. Taken together, these findings suggest that OEOA stimulates differentiation of K562 cells towards erythroid lineage, rather than megakaryocytic lineage.

### OEOA was Retained for a Longer Period in Mouse Blood Plasma

**Figure 7:**
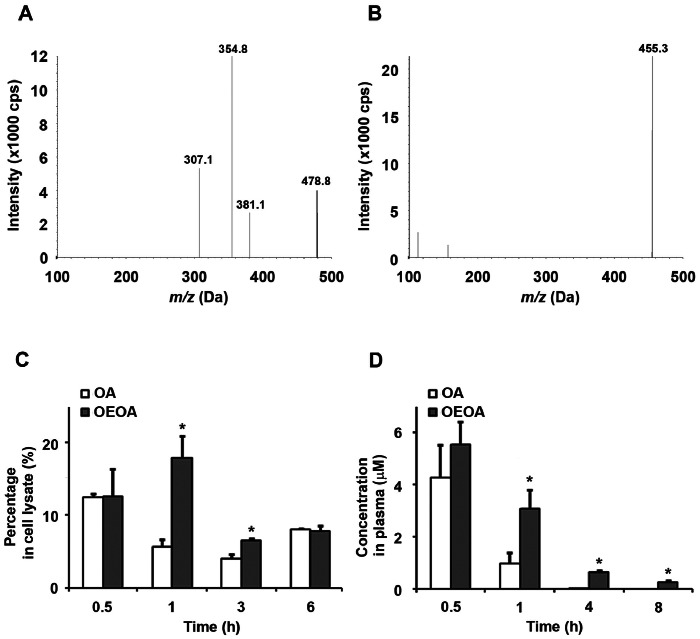
OEOA was retained for a longer period in mouse blood plasma than OA. MS-MS product ion mass spectra of OEOA (A) and OA (B). (C) K562 cells were treated with OA or OEOA (1 µM) for 6 h. Culture media and cell lysates were collected at indicated time points for HPLC-MS/MS analysis. Data was represented as mean ± SEM in cell lysate compared to total exposure, n = 2 (* *p*<0.05). (D) Concentrations of OA and OEOA in mouse plasma after intraperitoneal injection. Blood was collected from mice at different time points after single administration of OA and OEOA. Data was represented as mean ± SEM, n = 2 animals per time point (* *p*<0.05).

We next investigated whether the structural modification in OEOA altered the pharmacokinetic profile of the compound when compared with OA. First, we have developed the detection parameters of OEOA using HPLC-MS/MS analysis. Under the collision energy −70 V, the ion mass spectrum of OEOA was measured at *m/z* 478.8 with the daughter ions at *m/z* 381.1, 354.8, and 307.1 (Fig. 7A). Similar to the previous report [40], the product ion mass spectrum of OA was measured as *m/z* 455.3 (Fig. 7B). To investigate whether OEOA is able to pass through the plasma membrane and be detectable in the cellular compartment, K562 cells were incubated with OA or OEOA (1 µM) for various time intervals. Detection of OA or OEOA in culture medium and cell lysates was performed using HPLC-MS/MS analysis. Both OA and OEOA were readily detected in the cell lysates of K562 cells at 30 min after addition (Fig. 7C). Interestingly, the amount of OEOA was higher than OA in the cell lysates after 1 h-treatment. Next, we examined whether OEOA and OA could be detected in blood plasma of the mice after intraperitoneal administration (Fig. 7D). OEOA and OA (20 µmol/kg each) were co-administered to the same animal and simultaneously detected in plasma (OEOA: *m/z* 479 → 479, [M-H]^-^, *R_t_* 5.24 min; OA: *m/z* 455 → 455, [M-H]^-^, *R_t_* 3.69 min) using HPLC-MS/MS analysis. Quantitative analysis indicated that both OA and OEOA were readily detected in plasma at 30 min after injection (Fig. 7D). The concentration of OA and OEOA in plasma at *t_0.5_* was 4.25±1.277 and 5.51±0.938 µM, respectively. While the concentration of OA at *t_1_* and *t_4_* was 0.92±0.416 µM and 0.02±0.004 µM, OEOA remained at high level, 3.10±0.646 µM and 0.65±0.099 µM, respectively. At 8 h after administration, while the concentration of OA was below detection limit, the concentration of OEOA was 0.29±0.001 µM, suggesting that OEOA is retained in plasma for a longer period than OA.

## Discussion

OA and its derivatives have been shown to suppress the initiation and growth, as well as to induce differentiation and apoptosis of various tumors [12]–[18]. They also impact cancer progression by preventing angiogenesis, inhibiting metastasis and promoting anti-cancer immune responses [12], [16]–[18]. Despite these activities against solid tumors, OA is not effective in inhibiting the proliferation of leukemia cells. Recent progress has been made to improve the efficacy of OA against leukemia through generation of its derivatives [19], [41]. For example, a synthetic triterpenoid 2-cyano-3,12-dioxooleana-1,9-dien-28-oic acid (CDDO) induces differentiation and apoptosis in myeloid leukemia cells [20], [41], [42], while another synthetic oleanolic acid derivative N-[(3beta)-3-(acetyloxy)-28-oxoolean-12-en-28-yl]-glycine methyl ester (AOA-GMe) inhibits cell growth in K562 and B16 melanoma cells by inducing cell cycle arrest [19]. Here, we report that OEOA, an oxadiazole carboxylic acid derivative of OA, exhibits promising inhibitory effect on human leukemia cell proliferation. It is interesting to note that CDDO, AOA-GMe and OEOA all possess modification at C3 of OA, which supports the notion that the A-ring modification of OA forms a rational structural basis for future design of anti-leukemia drugs. Our findings further suggest that the structural modification in OEOA results in a longer duration of exposure when compared to OA. Whether the change in the pharmacokinetic profile of OEOA compared to OA is a result of slower metabolism or excretion and allows a longer duration of action remains to be determined.

In the present study, we provide molecular evidence to show that treatment of K562 cells with OEOA triggers cell cycle arrest in G1 phase. Mechanistically, we demonstrate that OEOA treatment significantly attenuated the expression levels of Cyclin D1, Cdk4 and Cdk6 in K562 cells, resulting in reduced phosphorylation of Rb and Cyclin E and accumulation of p27. In early G1 phase, Cyclin D binds to Cdk4 and/or Cdk6 to form cyclin-Cdk complexes, resulting in the activation of Cdks. Phosphorylation of Rb protein by the Cyclin D-Cdk4/6 complex in turn allows the expression of other cell cycle genes, such as Cyclin E. Association between Cyclin E and Cdk2 then leads to the phosphorylation and degradation of p27, allowing the transition from G1 to S phase [26], [27], [43]. The regulation of cyclins and Cdks expression by OEOA, together with the ability of OEOA to suppress RAMP expression, strongly suggest that OEOA inhibits proliferation of leukemia cells by modulating cell cycle protein expression.

Given that K562 cells are of CML origin, we also evaluated the potential effects of OEOA on CML by examining the expression of Bcr-Abl in K562 cells. The chimeric Bcr-Abl oncoprotein possesses a constitutive tyrosine kinase activity, which drives CML pathogenesis [35], [36], [44], and is the predominant therapeutic target in CML. Indeed, tyrosine kinase inhibitors, e.g. imatinib, have been successfully used for treating CML during chronic phase [8], [45], [46]. However, some CML patients become resistant to imatinib, especially in the blast crisis stage [8], [45], [46]. It has also been shown recently that the transforming capacity of Bcr-Abl could be independent of its kinase activity [47], further suggesting that inhibition of Bcr-Abl kinase activity is not sufficient to prevent CML development. Extensive efforts have therefore been made to search for new drugs and combinations to overcome imatinib resistance. In the present study, we have demonstrated that OEOA inhibits the protein expression, rather than targeting the kinase activity, of Bcr-Abl in K562 cells. Our findings raise the intriguing possibility of the potential application of OEOA, perhaps in combination with imatinib, to overcome imatinib resistance in treating CML.

Differentiation therapy has become one of the new strategies for treating leukemia, which induces maturation and eventual senescence of cancer stem cells, instead of killing them through cytotoxicity [16], [48]–[51]. In this context, it is noteworthy that OEOA does not induce cell death in the leukemia cells. Rather, it facilitates erythroid differentiation by inducing the expression of *γ-globin* and inhibiting Bcr-Abl expression and Erk1/2 activation in K562 cells. The beneficial role of OEOA on erythroid differentiation, especially to CML patients, may shed light on future leukemia drug development exploiting the differentiation approach.

In conclusion, our findings provide the first demonstration that a derivative of oleanolic acid, OEOA acts through unique mechanisms to inhibit leukemia cell proliferation by inducing G1 arrest and erythroid differentiation. Although further studies are required to determine the underlying target for its action, our results suggest OEOA may be a promising lead compound for leukemia treatment.
